# Plasma Sphingomyelin as a Post-Treatment Monitoring Biomarker for Pathological Response in Locally Advanced Rectal Cancer

**DOI:** 10.3390/cancers18132124

**Published:** 2026-06-30

**Authors:** Pedro Brandão, Lúcia Lacerda, Marisa D. Santos

**Affiliations:** 1 Colorectal Unit, Department of Surgery, Hospital de Santo António, Unidade Local de Saúde de Santo António (ULSSA), Largo Professor Abel Salazar, 4099-001 Porto, Portugal; marisasantos.cirurgia1@ulssa.min-saude.pt; 2 School of Medicine and Biomedical Sciences (ICBAS), University of Porto, 4050-313 Porto, Portugal; lucia.lacerda@ulssa.min-saude.pt; 3 Unit for Multidisciplinary Research in Biomedicine (UMIB), School of Medicine and Biomedical Sciences (ICBAS), University of Porto, Rua Jorge Viterbo Ferreira 228, 4050-313 Porto, Portugal; 4 ITR—Laboratory for Integrative and Translational Research in Population Health, Rua das Taipas 135, 4050-600 Porto, Portugal; 5 Laboratório de Bioquímica Genética, Serviço de Genética Laboratorial, Centro de Genética Médica Doutor Jacinto Magalhães, Clínica de Genética e de Patologia, Unidade Local de Saúde de Santo António (ULSSA), 4099-001 Porto, Portugal

**Keywords:** sphingomyelin, locally advanced rectal cancer, neoadjuvant chemoradiotherapy, biomarker, treatment response monitoring, tumour regression grade, metabolomics, organ preservation

## Abstract

**Background:** Organ-preservation strategies in locally advanced rectal cancer (LARC) increasingly require accurate assessment of response to neoadjuvant therapy, yet magnetic resonance imaging poorly distinguishes complete from near-complete responses. We investigated plasma sphingolipid dynamics across multiple treatment timepoints as monitoring biomarkers for pathological response. **Methods:** This single-centre cohort study included 86 patients with histologically confirmed LARC; 58 received neoadjuvant treatment followed by surgical resection with an evaluable Mandard tumour regression grade (TRG) and formed the response-evaluable cohort (19 good responders [TRG 1–2], 39 poor [TRG 3–5]). Plasma sphingomyelin (SM), sphingosine-1-phosphate and glucosylceramide were quantified by UPLC–MS/MS at baseline (M0), post-chemoradiotherapy (M1) and post-surgery (M2). Discrimination was assessed by receiver operating characteristic analysis and odds ratios, longitudinal trajectories by a linear mixed-effects model, and SM was compared head-to-head with serum carcinoembryonic antigen (CEA). **Results:** SM did not discriminate response at baseline (M0 AUC = 0.53) but did after treatment (M1 AUC = 0.750, *p* = 0.024; M2 AUC = 0.786, *p* = 0.010; pooled AUC = 0.767; odds ratio 10.67 at a 164.0 mg/L cutoff; negative predictive value 84%). SM correlated inversely with TRG grade (Spearman ρ = −0.43, *p* = 0.016) and outperformed serum CEA (AUC 0.74 vs. 0.60). Trajectories diverged over time (mixed-model time × group interaction *p* = 0.023) with no baseline difference (*p* = 0.96): good responders increased SM by +22.5% from M0 to M2, whereas poor responders declined by −8.4%. **Conclusions:** Plasma SM is not a pre-treatment predictor but emerges as a post-treatment monitoring biomarker for pathological response in LARC, supporting its integration into multimodal response assessment for organ-preservation decision-making. External validation in independent cohorts is warranted.

## Simple Summary

After chemoradiotherapy for locally advanced rectal cancer, clinicians must judge how completely the tumour has responded in order to choose between surgery and organ-preserving “watch-and-wait” strategies, yet current imaging often cannot distinguish a complete response from residual disease. We examined whether sphingomyelin, a lipid measured in blood, could help. In 58 patients, blood sphingomyelin did not differ between good and poor responders before treatment, so it cannot select patients in advance. After treatment, however, it was clearly higher in good responders, and increasingly so closer to surgery, tracking the degree of tumour regression, and it outperformed the standard blood marker CEA. Measuring sphingomyelin after treatment could therefore provide objective, inexpensive information to support response monitoring and organ-preservation decisions, complementing imaging. Independent studies are needed to confirm these findings.

## 1. Introduction

Locally advanced rectal cancer (LARC), defined as clinical stage T3–T4 and/or node-positive disease, affects approximately 40% of patients diagnosed with rectal cancer and represents a significant therapeutic challenge [1,2]. Standard treatment consists of neoadjuvant chemoradiotherapy (nCRT) followed by total mesorectal excision (TME), though total neoadjuvant therapy (TNT) strategies incorporating induction or consolidation chemotherapy have demonstrated improved outcomes in recent landmark trials [3,4]. Pathological complete response (pCR), observed in 15–27% of patients, is associated with excellent long-term survival and has emerged as a key endpoint driving treatment innovation [5].

The emergence of organ-preservation strategies, most notably the watch-and-wait (W&W) approach for patients achieving clinical complete response (cCR), has transformed the treatment landscape [6,7]. The International Watch & Wait Database has confirmed favourable long-term outcomes for carefully selected patients, with 5-year overall survival exceeding 85% [8]. Total neoadjuvant therapy has further expanded the proportion of patients eligible for organ preservation [9]. However, accurate response assessment remains the critical bottleneck: magnetic resonance imaging (MRI), the current standard for restaging, demonstrates suboptimal accuracy for distinguishing complete from near-complete responses, particularly in the presence of post-treatment fibrosis [10,11,12]. International consensus recommendations have emphasised the urgent need for complementary biomarkers to improve response assessment accuracy and support organ-preservation decision-making [13].

Circulating biomarkers offer the potential to complement imaging-based response assessment by providing objective, quantifiable molecular information reflecting systemic metabolic alterations induced by treatment. Metabolomics, the comprehensive analysis of small molecules in biological systems, has emerged as a powerful tool for biomarker discovery in oncology [14], with increasing applications in clinical practice [15]. Sphingolipids—a class of bioactive lipids including sphingomyelin (SM), ceramide, and sphingosine-1-phosphate (S1P)—play crucial roles in cancer biology through the sphingomyelin–ceramide pathway [16,17,18]. Ceramide, generated from SM by sphingomyelinase (SMase) activity, is a potent pro-apoptotic molecule that mediates radiation-induced cell death [19,20]. Conversely, S1P promotes cell survival through the “sphingolipid rheostat” mechanism, wherein the balance between pro-apoptotic ceramide and pro-survival S1P determines cell fate [21,22]. Alterations in sphingolipid metabolism have been demonstrated in colorectal cancer, with diagnostic and prognostic implications [23,24,25].

Despite this biological rationale, no study has systematically evaluated plasma sphingolipid dynamics across multiple treatment timepoints in LARC. Previous metabolomic studies in rectal cancer have primarily focused on tissue-based analyses or single-timepoint assessments. The MetLARC (Metabolic abnormalities on tumour response and resistance to neoadjuvant chemoradiotherapy in Locally Advanced Rectal Cancer) study was designed to address this gap through comprehensive metabolomic profiling at baseline, post-nCRT, and post-surgery. We hypothesised that plasma sphingolipid dynamics, rather than baseline levels, reflect treatment-induced tumour regression and could function as post-treatment monitoring biomarkers in LARC. Here, we test this hypothesis by characterising plasma sphingolipid trajectories across treatment timepoints and benchmarking their discriminatory performance against pathological response.

## 2. Materials and Methods

### 2.1. Study Design and Participants

**Figure 1:**
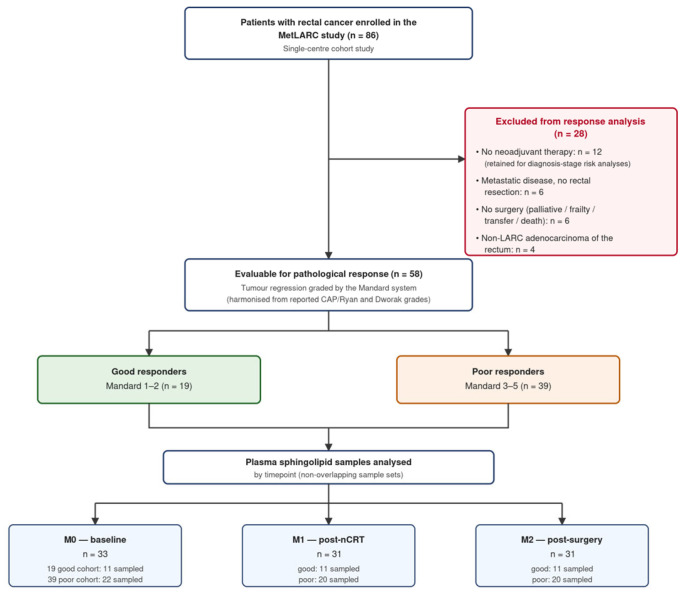
Study design and patient flow. Of 86 enrolled patients, 28 were excluded from the response analysis (no neoadjuvant therapy, n = 12 [retained for diagnosis-stage analyses]; metastatic disease without rectal resection, n = 6; no surgery owing to palliative intent, frailty, transfer, or death, n = 6; tumour not meeting LARC adenocarcinoma criteria, n = 4), leaving 58 patients with an evaluable Mandard tumour regression grade (TRG) (19 good [TRG 1–2]; 39 poor [TRG 3–5]). Plasma sphingomyelin was analysed at M0 (11 good/22 poor), M1 (11/20), and M2 (11/20); 15 patients had complete longitudinal sampling. TRG was harmonised to the Mandard system from grades originally reported using the College of American Pathologists/modified Ryan and Dworak systems.

The MetLARC study is a single-centre observational cohort study conducted at Unidade Local de Saúde de Santo António (ULSSA), Porto, Portugal. Patients with histologically confirmed locally advanced rectal cancer (cT3–T4 and/or cN+) treated with neoadjuvant therapy were studied; the cohort comprised patients enrolled prospectively together with additional patients whose banked plasma samples and clinical records were reviewed retrospectively. Inclusion criteria were age ≥ 18 years, Eastern Cooperative Oncology Group (ECOG) performance status 0–2, and adequate organ function. Patients who did not receive neoadjuvant therapy, did not undergo rectal resection, or whose tumour did not meet the criterion of rectal adenocarcinoma were not eligible for the response analysis (Figure 1). The study was approved by the Ethics Committee of ULSSA; all participants provided written informed consent, and the study was conducted in accordance with the Declaration of Helsinki and Good Clinical Practice guidelines.

### 2.2. Treatment Protocols

Patients received neoadjuvant treatment according to institutional protocols and individualised multidisciplinary tumour-board decisions. Treatment modalities included: (i) long-course chemoradiotherapy (50.4 Gy in 28 fractions with concurrent capecitabine 825 mg/m^2^ twice daily); (ii) short-course radiotherapy (25 Gy in five fractions), optionally followed by consolidation chemotherapy; and (iii) total neoadjuvant therapy (TNT) with induction or consolidation chemotherapy (CAPOX or FOLFOX) [3,4]. Surgery was performed 8–12 weeks after nCRT completion, with total mesorectal excision (TME) as the standard approach. Given the limited sample size per protocol subgroup, all treatment modalities were pooled for the primary analysis; this study is framed as exploratory and hypothesis-generating.

### 2.3. Sample Collection and Processing

Peripheral venous blood samples were collected at predefined timepoints: M0 (baseline, pre-treatment), M1 (post-nCRT, at restaging 8–10 weeks after treatment completion), and M2 (post-surgery, after TME). Blood was collected in EDTA tubes, centrifuged at 2214× *g* for 10 min at 4 °C within 4 h of collection, and plasma was aliquoted and stored at −70 °C until analysis. Dried blood spots (DBS) were prepared on Guthrie cards for acylcarnitine profiling and stored at 4 °C.

### 2.4. Metabolomic Profiling

All metabolomic analyses were performed at the Centro de Genética Médica Doutor Jacinto Magalhães (CGMJM), Unidade Local de Saúde de Santo António (ULSSA), Porto, Portugal, a certified clinical laboratory with validated quality control procedures. Sphingolipid quantification (SM, S1P, and glucosylceramide [GlcCer]) was performed by ultra-performance liquid chromatography–tandem mass spectrometry (UPLC-MS/MS) using an Acquity UPLC H-Class system coupled with a Xevo TQ-MS triple quadrupole mass spectrometer (Waters Corporation, Milford, MA, USA). Plasma extraction employed chloroform:methanol:formic acid, followed by vortexing for 30 s and centrifugation for 10 min at 11,300× *g*. Chromatographic separation used a Waters C18 BEH column (2.1 × 50 mm, 1.7 μm) at 50 °C, with a flow rate of 0.6 mL/min and an 8 min gradient; detection was performed in multiple reaction monitoring (MRM) mode [26]. Sphingolipid concentrations were expressed in mg/L. Each sphingolipid (SM, S1P and GlcCer) was quantified as a total class-level plasma concentration; individual molecular species (e.g., by acyl chain length) were not resolved. Amino acid profiling (n = 44) was conducted on deproteinised plasma by ion-exchange chromatography with post-column derivatization with ninhydrin, using a Biochrom 30+ Bio fully automated amino acid analyser (Biochrom Ltd., Cambridge, UK) with a five-buffer lithium citrate system. Acylcarnitine analysis (n = 33) was performed on DBS (3.2 mm punch) by UPLC–MS/MS. Amino acid and acylcarnitine concentrations were expressed in μmol/L.

### 2.5. Pathological Response Assessment

Pathological response was assessed on surgical specimens using the Mandard tumour regression grade (TRG) system [27]: TRG 1 (complete regression) through TRG 5 (no regression), categorised as good response (TRG 1–2) versus poor response (TRG 3–5). Where the original pathology report used an alternative regression system, the grade was converted to the equivalent Mandard category using a pre-specified mapping (College of American Pathologists/modified Ryan 0→Mandard 1, 1 → 2, 2 → 3, 3 → 4–5; Dworak 4 → Mandard 1, 3 → 2, 2 → 3, 1 → 4, 0 → 5). Assessment was performed by experienced gastrointestinal pathologists. The recognised interobserver and inter-system variability of regression grading [28] is itself one of the limitations that an objective circulating biomarker could help mitigate.

### 2.6. Statistical Analysis

Continuous variables are presented as mean ± standard deviation. Comparisons between response groups used the Mann–Whitney U test. Discriminatory performance was assessed by receiver operating characteristic (ROC) analysis, reporting the area under the curve (AUC) with sensitivity and specificity at the optimal Youden index cutoff; odds ratios were derived at that cutoff. The Spearman rank correlation coefficient assessed the relationship between SM levels and Mandard TRG grade. To accommodate the unbalanced longitudinal sampling structure (15 patients with complete M0–M2 observations), a linear mixed-effects model with random patient intercepts was fitted to all available SM observations, modelling SM as a function of timepoint, response group, and their interaction. Plasma SM was compared head-to-head with serum carcinoembryonic antigen (CEA)—available as pre-treatment and post-treatment measurements—using ROC analysis with asymptotic 95% confidence intervals, Spearman correlation, and a combined logistic-regression model. The exploratory screen of 44 amino acids and 33 acylcarnitines was corrected for multiple testing using the Benjamini–Hochberg false discovery rate [29]. Statistical significance was *p* < 0.05 (two-sided). Statistical analyses were performed using IBM SPSS Statistics version 31.0 (IBM Corp., Armonk, NY, USA).

## 3. Results

### 3.1. Patient Characteristics

A total of 86 unique patients with histologically confirmed LARC were enrolled in the MetLARC study (Figure 1). Twenty-eight patients were excluded from the response analysis: 12 did not receive neoadjuvant therapy (retained only for diagnosis-stage analyses), six had metastatic disease without rectal resection, six did not undergo surgery (palliative intent, frailty, transfer to another institution, or early death), and four had a tumour not meeting the criterion of rectal adenocarcinoma. The remaining 58 patients constituted the response-evaluable cohort: 19 (32.8%) good responders (Mandard TRG 1–2) and 39 (67.2%) poor responders (TRG 3–5). Plasma sphingomyelin was measured at baseline (M0; 11 good/22 poor), post-nCRT (M1; 11 good/20 poor), and post-surgery (M2; 11 good/20 poor); 15 patients had matched samples across all three timepoints. Baseline characteristics were well balanced between response groups (Table 1).

### 3.2. Baseline Sphingomyelin: Absence of Predictive Capacity

**Figure 2:**
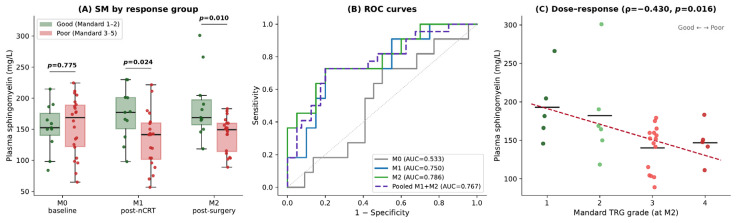
Plasma sphingomyelin as a post-treatment monitoring biomarker. (**A**) Plasma SM in good versus poor responders at M0 (*p* = 0.775), M1 (*p* = 0.024), and M2 (*p* = 0.010). (**B**) ROC curves: M0 (AUC = 0.53), M1 (AUC = 0.750), M2 (AUC = 0.786), pooled post-treatment (AUC = 0.767). (**C**) SM by individual Mandard TRG grade at M2, showing the inverse dose–response gradient (ρ = −0.43, *p* = 0.016). Good responders in green, poor responders in red.

At baseline (M0), plasma SM levels did not differ between response groups (good: 152.7 ± 38.4 mg/L, n = 11; poor: 154.9 ± 46.5 mg/L, n = 22; *p* = 0.775; AUC = 0.53; Figure 2A,B). S1P (*p* = 0.504) and GlcCer (*p* = 0.236) likewise showed no baseline discrimination. These findings establish that SM does not function as a predictive (pre-treatment) biomarker in this cohort. Across post-treatment timepoints, neither S1P nor GlcCer discriminated response (all *p* > 0.05) or correlated with tumour regression grade, in contrast to SM (Supplementary Table S2), supporting the specificity of the SM signal. Although ceramide glucosylation is biologically linked to treatment resistance [16,18], baseline GlcCer did not significantly discriminate response (AUC ≈ 0.63), consistent with the absence of a baseline SM difference.

### 3.3. Post-nCRT Sphingomyelin Discrimination (M1)

Following neoadjuvant treatment, significant SM discrimination emerged. At M1, good responders had significantly higher plasma SM than poor responders (173.2 ± 42.1 vs. 133.1 ± 45.1 mg/L; *p* = 0.024; Figure 2A). ROC analysis yielded an AUC of 0.750, with an optimal cutoff of 164.0 mg/L (sensitivity 72.7%, specificity 80.0%; odds ratio 10.67). The SM/S1P ratio further enhanced discrimination at M1 (good: 545.5 ± 235.2 vs. poor: 357.3 ± 108.0; *p* = 0.016), consistent with the sphingolipid rheostat hypothesis.

### 3.4. Post-Surgery Sphingomyelin Discrimination (M2)

Discrimination strengthened at the post-surgical timepoint. Good responders maintained significantly higher SM levels (187.0 ± 53.5 vs. 141.9 ± 28.0 mg/L; *p* = 0.010; Figure 2A). ROC analysis demonstrated an AUC of 0.786 (cutoff 164.5 mg/L; sensitivity 72.7%, specificity 80.0%; odds ratio 10.67), a progressive improvement from M1.

### 3.5. Pooled Post-Treatment Analysis

Combining all post-treatment observations (M1 + M2) confirmed robust discrimination (Table 2): good responders (180.1 ± 47.5 mg/L, n = 22) versus poor responders (137.5 ± 37.3 mg/L, n = 40; *p* = 0.0006; AUC = 0.767). The pooled odds ratio at a 164.0 mg/L cutoff was 10.67 (sensitivity 72.7%, specificity 80.0%), with a negative predictive value of 84.2%—directly relevant to excluding incomplete response in watch-and-wait patient selection. This temporal reinforcement supports the biological plausibility of SM as a treatment-response monitoring marker. The 164.0 mg/L cutoff was derived from the present dataset and is exploratory, requiring external validation before use as a clinical decision threshold. Because predictive values depend on response prevalence, the reported negative predictive value reflects this cohort’s response rate and may differ in populations with different response rates.

### 3.6. TRG Dose–Response Gradient

SM levels at M2 demonstrated a significant inverse dose–response relationship with Mandard TRG grade (Figure 2C): TRG 1 (192.8 ± 46.3 mg/L, n = 5), TRG 2 (182.2 ± 62.9 mg/L, n = 6), TRG 3 (140.2 ± 29.3 mg/L, n = 15), and TRG 4 (146.9 ± 25.6 mg/L, n = 5). The Spearman correlation coefficient was ρ = −0.43 (*p* = 0.016), indicating that SM elevation is not a binary phenomenon but exhibits a graded relationship with the degree of pathological regression.

### 3.7. Divergent Longitudinal Trajectories

**Figure 3:**
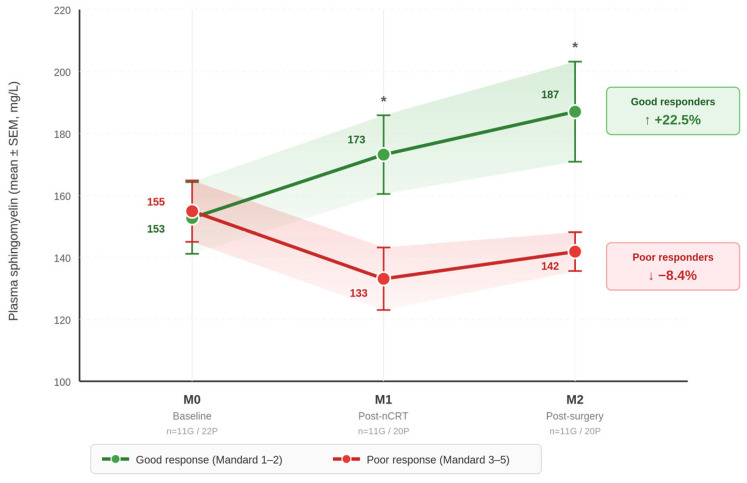
Divergent longitudinal sphingomyelin trajectories. Mean plasma SM (±SEM) from M0 through M1 to M2 by response group. Good responders (green) show progressive elevation (+22.5%), poor responders (red) decline overall (−8.4%). A linear mixed-effects model confirmed a significant timepoint × group interaction (β = +21.3 mg/L per timepoint, *p* = 0.023) with no baseline difference (*p* = 0.96).

Longitudinal analysis revealed divergent trajectories between response groups (Figure 3). Good responders showed progressive SM elevation from M0 (152.7 mg/L) through M1 (173.2 mg/L) to M2 (187.0 mg/L), a cumulative increase of +22.5%, whereas poor responders declined overall from M0 (154.9 mg/L) through M1 (133.1 mg/L) to M2 (141.9 mg/L), a net change of −8.4%. To accommodate the unbalanced longitudinal sampling structure, a linear mixed-effects model with random patient intercepts was fitted to all available SM observations. The model confirmed the principal finding: there was no difference between groups at baseline (group effect *p* = 0.96), but good responders increased their SM significantly more than poor responders over time (time × group interaction β = +21.3 mg/L per timepoint, *p* = 0.023).

### 3.8. Exploratory Multi-Pathway Screening

As an exploratory screen, 77 additional metabolites (44 amino acids and 33 acylcarnitines) were compared between response groups at M1. Glutamine (565.5 ± 63.3 vs. 502.9 ± 90.7 μmol/L; *p* = 0.029) and ammonia (141.2 ± 27.3 vs. 187.6 ± 54.9 μmol/L; *p* = 0.033) showed nominal elevation and reduction, respectively, in good responders. However, after Benjamini–Hochberg correction for multiple testing, no metabolite remained significant (all q ≈ 0.88; Supplementary Table S1). These observations are therefore hypothesis-generating only; the principal finding rests on plasma sphingomyelin.

### 3.9. Comparison with Serum CEA

To assess the added value of plasma SM over serum CEA, a head-to-head comparison was performed (Table 3). At baseline, neither marker discriminated pathological response (SM at M0: AUC = 0.53, n = 33; pre-treatment CEA: AUC = 0.535, 95% CI 0.37–0.70, n = 51). After treatment, plasma SM discriminated response substantially better than CEA (SM: AUC = 0.740, 95% CI 0.58–0.90, n = 44; post-treatment CEA: AUC = 0.602, 95% CI 0.43–0.78, n = 46). Plasma SM and serum CEA were essentially uncorrelated in post-treatment samples (Spearman ρ = −0.08, *p* = 0.61); a combined SM + CEA logistic model did not improve discrimination over SM alone (combined AUC = 0.744 vs. 0.740). Overall, plasma SM outperformed serum CEA for post-treatment response discrimination, supporting its role as an objective, quantitative adjunct rather than a redundant measurement.

## 4. Discussion

This single-centre cohort study demonstrates that plasma sphingomyelin functions as a dynamic post-treatment monitoring biomarker for pathological response assessment in LARC, with progressive discriminatory capacity from post-nCRT (AUC = 0.750) to post-surgery (AUC = 0.786), a significant inverse dose–response relationship with the Mandard tumour regression grade (ρ = −0.43, *p* = 0.016), and divergent longitudinal trajectories confirmed by a linear mixed-effects model (time × group interaction *p* = 0.023; no baseline difference, *p* = 0.96). To our knowledge, this is the first study to systematically evaluate plasma sphingolipid dynamics across multiple treatment timepoints in LARC.

### 4.1. Biological Rationale

The observed pattern of elevated post-treatment SM in good responders has a robust biological basis in the sphingomyelinase–ceramide pathway of radiation-induced apoptosis [19,20]. Ionising radiation activates acid sphingomyelinase (ASMase), which hydrolyses membrane SM to generate ceramide—a potent pro-apoptotic second messenger [16,19]. In tumours with effective radiation response, active ceramide generation from SM is followed by systemic metabolic compensation: hepatic SM synthesis is upregulated to replenish depleted membrane SM pools, resulting in elevated circulating SM. Conversely, in radiation-resistant tumours, limited ceramide generation produces no compensatory SM synthesis stimulus, and plasma SM remains low or declines. This “ceramide sink” model is supported by the sphingolipid rheostat concept [17,21,22]; our observation that the SM/S1P ratio enhanced discrimination at M1 (*p* = 0.016) is consistent with effective treatment shifting the rheostat toward ceramide-mediated apoptosis. We emphasise that, in the absence of tumour-tissue sphingolipid measurements or enzyme-activity assays, this sphingomyelinase–ceramide mechanism remains hypothetical and is offered as a plausible interpretation of the plasma observations.

### 4.2. Clinical Significance of the Monitoring Paradigm

The distinction between a predictive biomarker (pre-treatment) and a monitoring biomarker (post-treatment) is clinically fundamental. Our data demonstrate that SM does not predict pathological response before treatment begins (M0: *p* = 0.775, AUC = 0.53) and therefore should not be used for pre-treatment patient selection. However, SM emerges as a significant discriminator after treatment, with progressively stronger performance (M1: *p* = 0.024, AUC = 0.750; M2: *p* = 0.010, AUC = 0.786). This monitoring paradigm is clinically critical because MRI shows well-documented limitations in distinguishing complete from near-complete responses, particularly in the presence of treatment-induced fibrosis [10,11,12], and international consensus has called for complementary biomarkers [13]. The pooled post-treatment odds ratio of 10.67 at a cutoff of 164.0 mg/L indicates that patients above this threshold are approximately eleven times more likely to be good responders; combined with a negative predictive value of 84%, this suggests clinical utility in excluding incomplete responses. Beyond complementary biological information, an objective, quantitative plasma biomarker may help mitigate two well-documented sources of subjectivity in current LARC response assessment: the interobserver variability of pathological TRG grading [28] and the misclassification of complete responders by post-treatment MRI [30]. From a clinical standpoint, the actionable timepoint is M1 (after chemoradiotherapy, before surgery), when organ-preservation and watch-and-wait decisions are made; the post-surgical timepoint (M2) cannot inform such decisions and is reported because it reinforces the biological association between SM and pathological response.

### 4.3. TRG Gradient and Dose–Response Relationship

The significant inverse correlation between SM levels and TRG grade (ρ = −0.43, *p* = 0.016) provides important biological validation. Rather than a binary phenomenon, SM elevation demonstrates a graded response proportional to the degree of pathological regression, suggesting that SM could contribute not only to binary response classification but also to continuous assessment of regression quality [13].

### 4.4. Multi-Pathway Metabolic Reprogramming

Beyond sphingolipid metabolism, exploratory screening identified nominal alterations in glutamine (*p* = 0.029) and ammonia (*p* = 0.033), with weaker trends in long-chain acylcarnitines and amino acids [31,32,33]. However, none of these signals survived correction for multiple testing across the 77 metabolites screened (Benjamini–Hochberg q ≈ 0.88; Supplementary Table S1). They are therefore presented as exploratory, hypothesis-generating observations, and the principal conclusions rest on plasma sphingomyelin.

### 4.5. Comparison with Existing Literature

Our findings extend the literature on sphingolipid alterations in colorectal cancer. Pan et al. [23] demonstrated the diagnostic value of serum sphingolipids in CRC, whilst Markowski et al. [24,25] showed that ceramide profiles correlate with disease stage and that sphingolipid profiles distinguish rectal from colon cancer. These studies primarily examined diagnostic rather than treatment-monitoring applications, and analysed tissue rather than circulating biomarkers. Our peripheral blood analysis captures systemic metabolic consequences of treatment; elevated circulating SM in good responders is consistent with enhanced hepatic SM synthesis to compensate for ceramide-mediated SM depletion in responsive tumour tissue [16,19]. Jia et al. [34] used untargeted metabolomics to identify serum biomarkers predictive of response to nCRT; our study contributes by focusing on sphingolipid dynamics and demonstrating the importance of multi-timepoint assessment.

### 4.6. Positioning of SM in the Multimodal Response Assessment Landscape

Our head-to-head analysis indicates that plasma SM outperforms serum CEA for post-treatment response discrimination (SM AUC = 0.740 vs. CEA AUC = 0.602). CEA discriminated pathological response poorly at both baseline and post-treatment, consistent with its established limited sensitivity for incomplete response after nCRT [35]. Plasma SM and serum CEA were essentially uncorrelated post-treatment (ρ = −0.08), indicating biologically distinct dimensions; however, a combined model did not improve on SM alone (AUC 0.744 vs. 0.740). Plasma SM is therefore best positioned as a superior single monitoring measurement rather than an additive complement to CEA. In the broader landscape, MRI remains limited by post-treatment fibrosis [10,11,12]; ctDNA demonstrates high specificity but limited sensitivity for minimal residual disease [36]; and CEA shows low sensitivity for incomplete response. Plasma SM offers an objective, quantitative, inexpensive measurement that may strengthen multimodal restaging. Although direct comparison with ctDNA was not possible, the orthogonality of SM relative to CEA supports the hypothesis that it may also complement ctDNA-based assessment—a question warranting prospective evaluation. We emphasise that plasma SM is intended to complement existing multimodal response assessment rather than to replace established markers such as CEA.

### 4.7. Strengths and Limitations

Strengths include pre-specified multi-timepoint sampling; comprehensive metabolomic coverage across three pathways; validated analytical methodologies in a certified clinical laboratory; standardised pathological response assessment harmonised to the Mandard TRG system; and consistent results across multiple statistical approaches, including a linear mixed-effects model. Limitations must be acknowledged. First, this is a single-centre study of moderate size (58 response-evaluable patients), powered for discovery rather than validation, with per-timepoint subgroups of 11 good/22 poor (M0), 11/20 (M1), and 11/20 (M2). Second, the cohort combined prospectively and retrospectively collected samples, and tumour regression grades originally reported in different systems (College of American Pathologists/modified Ryan, Dworak) were harmonised to the Mandard scale; although performed conservatively, residual misclassification cannot be fully excluded. Third, the non-overlapping sampling limited complete longitudinal data to 15 patients, addressed using a mixed-effects model. Fourth, the heterogeneous neoadjuvant regimens, allocated by individualised multidisciplinary decisions, support the exploratory framing. Fifth, exploratory multi-pathway associations did not survive multiple-testing correction. Finally, external validation in an independent multicentre cohort is required before clinical implementation. Moreover, because the cohort pooled heterogeneous neoadjuvant regimens (short-course radiotherapy-based TNT and conventional long-course chemoradiotherapy), the results should not be assumed to apply equally across all regimens; the study was not powered for subgroup analysis or to establish regimen independence.

### 4.8. Future Directions

External validation should be prioritised, ideally in a multicentre cohort with standardised sample processing. Integration of SM into multimodal algorithms combining MRI, ctDNA, and metabolomic biomarkers could achieve the accuracy required for confident organ-preservation decisions. Mechanistic studies using in vitro models of radiation response would strengthen the biological foundation, and multi-metabolite panels incorporating SM may further improve discriminatory performance.

## 5. Conclusions

Plasma sphingomyelin does not function as a pre-treatment predictor of pathological response and therefore lacks utility for upfront patient selection in LARC. However, SM emerges as a robust post-treatment monitoring biomarker, demonstrating progressive discriminatory capacity from post-nCRT (AUC = 0.750) to post-surgery (AUC = 0.786), a significant inverse dose–response relationship with TRG grade (ρ = −0.43, *p* = 0.016), and divergent longitudinal trajectories between response groups (+22.5% in good vs. −8.4% in poor responders; linear mixed-model time × group interaction *p* = 0.023). Given the documented limitations of post-treatment MRI, plasma SM addresses an unmet clinical need by providing objective, quantitative information complementary to imaging-based restaging. These findings support integration of SM measurement into multimodal response assessment strategies for organ-preservation decision-making in LARC. External validation in an independent multicentre cohort is warranted.

## Figures and Tables

**Table 1:** Baseline characteristics of the response-evaluable cohort by treatment response.

Characteristic	Total (N = 58)	Good (n = 19)	Poor (n = 39)	*p*
Age at diagnosis, y (mean ± SD)	66.3 ± 11.7	64.0 ± 10.4	67.5 ± 12.2	0.205
Male sex, n (%)	35 (60.3)	8 (42)	27 (69)	0.085
Clinical T3, n (%)	43 (74.1)	14 (74)	29 (74)	1.000
Clinical T4, n (%)	11 (19.0)	5 (26)	6 (15)	0.476
Clinical N+, n (%)	44 (75.9)	16 (84)	28 (72)	0.350
TNT protocol, n (%)	10 (17.2)	6 (32)	4 (10)	0.065
CEA, ng/mL (median, IQR) *	3.4 (2.2–8.9)	3.2 (2.2–9.4)	3.7 (2.2–7.4)	0.686
SM at M0, mg/L (mean ± SD) †	154.2 ± 43.4	152.7 ± 38.4	154.9 ± 46.5	0.775
S1P at M0, mg/L †	0.519 ± 0.180	0.489 ± 0.181	0.533 ± 0.182	0.504
GlcCer at M0, mg/L †	0.093 ± 0.027	0.098 ± 0.027	0.091 ± 0.027	0.236

*p*-values: Mann–Whitney U test (continuous), Fisher’s exact test (categorical). * CEA available for 51/58 patients. † Baseline sphingolipids measured in the M0 subset (11 good/22 poor). Body mass index is not reported (height/weight not recorded in the source database).

**Table 2:** Diagnostic performance of plasma sphingomyelin by timepoint.

Timepoint	n (G/P)	AUC	Cutoff (mg/L)	Sens (%)	Spec (%)	OR	*p*
M0 (baseline)	11/22	0.53	–	–	–	–	0.775
M1 (post-nCRT)	11/20	0.750	164.0	72.7	80.0	10.67	0.024
M2 (post-surgery)	11/20	0.786	164.5	72.7	80.0	10.67	0.010
Pooled (M1 + M2)	22/40	0.767	164.0	72.7	80.0	10.67	0.0006

Pooled negative predictive value 84.2%. AUC, area under the ROC curve; OR, odds ratio (at the Youden cutoff); G, good; P, poor responders.

**Table 3:** Head-to-head discrimination of pathological response: plasma SM versus serum CEA.

Phase	Plasma SM—AUC (95% CI), n	Serum CEA—AUC (95% CI), n
Baseline (pre-treatment)	0.53, n = 33	0.535 (0.37–0.70), n = 51
Post-treatment	0.740 (0.58–0.90), n = 44	0.602 (0.43–0.78), n = 46

AUC for good pathological response (TRG 1–2). 95% CI by the asymptotic (Hanley–McNeil) method. SM post-treatment computed as the patient-level mean of available M1/M2 values (pooled per-observation AUC 0.767). Post-treatment SM vs. CEA: Spearman ρ = −0.08, *p* = 0.61; combined SM + CEA AUC = 0.744.

## Data Availability

The data presented in this study are available from the corresponding author upon reasonable request.

## Supplementary Materials

The following supporting information can be downloaded at: https://www.mdpi.com/article/10.3390/cancers18132124/s1, Table S1, exploratory metabolite screening at M1 (good vs. poor responders) with Benjamini–Hochberg false discovery rate correction. Table S2, specificity of the sphingomyelin signal across the targeted sphingolipid panel (SM, S1P and GlcCer) by timepoint.

## Abbreviations

The following abbreviations are used in this manuscript:

ASMase	acid sphingomyelinase
AUC	area under the curve
CEA	carcinoembryonic antigen
CI	confidence interval
cCR	clinical complete response
CRC	colorectal cancer
ctDNA	circulating tumour DNA
DBS	dried blood spots
ECOG	Eastern Cooperative Oncology Group
FDR	false discovery rate
GlcCer	glucosylceramide
LARC	locally advanced rectal cancer
MRI	magnetic resonance imaging
MRM	multiple reaction monitoring
nCRT	neoadjuvant chemoradiotherapy
NPV	negative predictive value
OR	odds ratio
pCR	pathological complete response
ROC	receiver operating characteristic
S1P	sphingosine-1-phosphate
SM	sphingomyelin
SMase	sphingomyelinase
TME	total mesorectal excision
TNT	total neoadjuvant therapy
TRG	tumour regression grade
UPLC-MS/MS	ultra-performance liquid chromatography–tandem mass spectrometry
W&W	watch-and-wait.
