# Supplementary material for: Plasma Sphingomyelin as a Post-Treatment Monitoring Biomarker for Pathological Response in Locally Advanced Rectal Cancer
Source: Cancers (Basel). 2026 Jun 30;18(13):2124. doi: 10.3390/cancers18132124 (PMC13359493; doi:10.3390/cancers18132124)
Supplement: Supplementary file 1 [file cancers-18-02124-s001.zip › cancers-4385227-supplementary.pdf]

Supplementary Material

Plasma sphingomyelin as a post-treatment monitoring biomarker for pathological response in locally advanced rectal cancer

Supplementary Table S1. Exploratory metabolite screening at M1 (good vs poor responders), with Benjamini–Hochberg false discovery rate correction.

Metabolite	Pathway	Good (mean)	Poor (mean)	p (raw)	q (BH-FDR)
Glutamine, µmol/L	Amino acid	565.5	502.9	0.029	0.88
Ammonia, µmol/L	Nitrogen	141.2	187.6	0.033	0.88
Histidine, µmol/L	Amino acid	72.5	61.7	0.063	0.88
Phosphoserine, µmol/L	Amino acid	4.5	8.5	0.065	0.88
Serine, µmol/L	Amino acid	114.2	96.8	0.072	0.88
C5:1, µmol/L	Acylcarnitine	0.013	0.004	0.075	0.88
C18, µmol/L	Acylcarnitine	0.568	0.466	0.103	0.88

Response-evaluable cohort, M1 timepoint (11 good / 20 poor). Mann–Whitney U test; q-values from the Benjamini–Hochberg false discovery rate across all 77 metabolites tested (44 amino acids, 33 acylcarnitines). Of 77 metabolites, 2 reached nominal p<0.05 and none survived FDR correction. Full screening data are available from the corresponding author upon reasonable request.

Supplementary Table S2. Specificity of the sphingomyelin signal across the targeted sphingolipid panel.

Discrimination of pathological response (good [Mandard TRG 1–2] vs. poor [TRG 3–5]) for each plasma sphingolipid at baseline (M0), post-chemoradiotherapy (M1) and post-surgery (M2) in the response-evaluable cohort (n = 58; 19 good, 39 poor). AUC, area under the ROC curve; p, Mann–Whitney U test; ρ, Spearman correlation with Mandard TRG (pooled post-treatment, M1+M2).

Analyte	M0 AUC (p)	M1 AUC (p)	M2 AUC (p)	ρ vs TRG (p)
**Sphingomyelin (SM)**	0.47 (0.78)	0.75 (0.024)	0.79 (0.010)	−0.41 (0.001)
**Sphingosine-1-phosphate (S1P)**	0.43 (0.50)	0.41 (0.42)	0.36 (0.22)	+0.06 (0.65)
**Glucosylceramide (GlcCer)**	0.63 (0.24)	0.53 (0.77)	0.51 (0.92)	−0.05 (0.72)

Among the three sphingolipids quantified, only SM discriminated pathological response and correlated inversely with tumour regression grade; S1P and GlcCer showed no discrimination at any timepoint and no correlation with TRG, supporting the specificity of the SM finding.
